# Asporin, a susceptibility gene in osteoarthritis, is expressed at higher levels in the more degenerate human intervertebral disc

**DOI:** 10.1186/ar2660

**Published:** 2009-03-27

**Authors:** Helen E Gruber, Jane A Ingram, Gretchen L Hoelscher, Natalia Zinchenko, Edward N Hanley, Yubo Sun

**Affiliations:** 1 Department of Orthopedic Surgery, Carolinas Medical Center, PO Box 32861, Charlotte, NC 28232, USA

## Abstract

**Introduction:**

Asporin, also known as periodontal ligament-associated protein 1 (PLAP1), is a member of the family of small leucine-rich proteoglycan (SLRP) family. It is present within the cartilage extracellular matrix (ECM), and is reported to have a genetic association with osteoarthritis. Its D14 allele has recently been found to be associated with lumbar disc degeneration in Asian subjects. There have been no studies, however, of this gene's normal immunohistochemical localization within the human intervertebral disc, or of expression levels in Caucasian individuals with disc degeneration.

**Methods:**

Studies were approved by our human subjects Institutional Review Board. Methods included immunohistochemical localization of asporin in the disc of humans and the sand rat (a small rodent with spontaneous age-related disc degeneration), and Affymetrix microarray analysis of asporin gene expression *in vivo *and *in vitro*.

**Results:**

Immunohistochemical studies of human discs revealed that some, but not all, cells of the outer annulus expressed asporin. Fewer cells in the inner annulus contained asporin, and it was rarely present in cells in the nucleus pulposus. Similar patterns were found for the presence of asporin in lumbar discs of sand rats. Substantial relative gene expression levels were seen for asporin in both disc tissue and in annulus cells grown in three-dimensional culture. More degenerate human discs (Thompson grade 4) showed higher expression levels of asporin than did less degenerate (grade 1, 2 and 3) discs, *P *= 0.004.

**Conclusions:**

In the discs of Caucasian subjects studied here, and in the sand rat, greater immunolocalization levels were found in the outer compared to inner annulus. Localization was rare in the nucleus. Gene expression studies showed greatest expression of asporin in the more degenerate human discs *in vivo*.

## Introduction

Asporin, also known as periodontal ligament-associated protein 1 (PLAP1), is an interesting, recently discovered leucine-rich protein that is a member of the family of small leucine-rich proteoglycan (SLRP) family and is associated with the extracellular matrix (ECM) in cartilage, meniscus and several other tissues [1,2]. The normal asporin allele contains 13 aspartic acid repeats in a 382 amino acid protein, and is designated D13. There are now three polymorphisms thought to be strongly associated with osteoarthritis (OA) susceptibility; these occur in the asporin gene (*ASPN*), the secreted frizzled-related protein 3 gene (*FRZB*), and the calmodulin 1 gene (*CALM1*) [3].

Recent studies have identified populations of individuals with osteoarthritis of the knee and asporin alleles with 14 aspartic acid repeats in the N-terminal region of the protein (designated D14). Work by Kizawa *et al*. first identified an association of the *ASPN *single nucleotide polymorphisms (SNPs) with knee and hip OA in Japanese patients [4]. As shown by Iida *et al*., there are at least 19 SNPs in asporin in Japanese patients with OA [5]. Associations of the *ASPN *SNPs with OA have been studied by Shi *et al*. [6] and by Nakamura *et al*. [7].

In non-Asian populations, however, recent studies appear to show a lack of association of the *ASPN *SNPs with OA susceptibility, as reflected in the work by Rodriguez-Lopez *et al*. in Spanish Caucasians [8] and work by Mustafa *et al*. in British Caucasians [9].

Our laboratory has become interested in asporin in the human intervertebral disc following the finding of Song *et al*. of an association of the *ASPN *D14 allele with disc degeneration in Asians [10]. Our literature search was not able to identify any studies showing immunolocalization of asporin in the human disc. The objectives of the present study, therefore, were to determine the localization patterns of asporin within the human and sand rat intervertebral disc, and to assess asporin expression in the human disc *in vivo *and *in vitro*.

## Materials and methods

### Clinical study population

Experimental study of human disc specimens was approved prospectively by the authors' Human Subjects Institutional Review Board at Carolinas Medical Center. The need for informed consent was waived since disc tissue was removed as part of routine surgical practice. Scoring of disc degeneration utilized the Thompson scoring system; this system scores disc degeneration over the spectrum from a healthy disc (Thompson grade I) to discs with advanced degeneration (grade V, the most advanced stage of degeneration) [11]. Patient specimens were derived from surgical disc procedures performed on individuals with herniated discs and degenerative disc disease. Surgical specimens were transported to the laboratory in sterile tissue culture medium. Care was taken to remove all granulation tissue and to sample only disc tissue. Non-surgical control donor disc specimens were obtained via the National Cancer Institute Cooperative Human Tissue Network (CHTN); they were shipped overnight to the laboratory in sterile tissue culture medium and processed as described below. Specimen procurement from the CHTN was included in our approved protocol by our human subjects Institutional Review board.

### Sand rat intervertebral disc tissue

Animal studies were carried out following approval by our Institutional Animal Care and Use Committee. *Psammomys obesus*, the sand rat, is studied in our laboratory as a model of spontaneous, age-related disc degeneration. Colony housing and animal diet have been previously described [12,13]. Spines from seven animals were analyzed in the present study of immunolocalization of asporin. Lumbar spines were removed immediately after rats were killed, fixed in 10% neutral buffered formalin, decalcified, and embedded in paraffin. Discs were individually embedded as mid-sagittal sections and also as *en face *sections individual discs. Sections were processed as described below for immunohistochemical studies.

### Expression of asporin *in vivo *and *in vitro*

Analysis of human disc tissue was carried out as previously described using laser capture microdissection methods [14]. Cells cultured in three dimensions and in monolayers were assayed for gene expression using the Affymetrix microarray system (Affymetrix, Santa Clara, CA, USA). Total RNA was extracted from cells using the TRIzol reagent (Gibco, Carlsbad, CA, USA), reverse transcribed to double-stranded cDNA, subjected to two rounds of transcription, and hybridized to the DNA microarray in the Affymetrix Fluidics Station 400. Affymetrix human U133 X3P arrays were used. The GCOS Affymetrix GeneChip Operating System (version 1.2, Affymetrix) was used for determining gene expression levels of asporin [GenBank:NM_017680.1]. Gene array data have been uploaded to the Gene Expression Omnibus database [GEO:GSE15227].

### Immunolocalization of asporin

Slides were deparaffinized in xylene and hydrated through graded alcohols to distilled water. The remainder of the procedure was performed using the Dako AutostainerPlus (Dako, Carpenteria, CA, USA). Endogenous peroxidase was blocked using 3%H_2_O_2 _(Sigma, St Louis, MO, USA). Slides were incubated for 1 h with polyclonal anti-asporin (GenWay Biotech, San Diego, CA, USA) at a 1:400 dilution. Secondary antibody was LSAB2 Link Antibody (Dako) applied for 10 minutes followed by peroxidase-conjugated streptavidin (Dako) for 10 minutes, and DAB (Dako) for 5 minutes. Some sand rat spine specimens and three-dimensional cultured human disc cells were prepared using the Vector VIP (Vector Laboratories, Burlingame, CA, USA) red chromagen. Universal Negative Control Rabbit (Dako) was used as a negative control. A positive control from a young rat articular cartilage joint was also included with each run. Slides were removed from the stainer, rinsed in water, counterstained with light green, dehydrated, cleared and mounted with resinous mounting media.

### Three-dimensional culture of human annulus cells

Human annulus cells were cultured in monolayer or three-dimensional culture in a collagen sponge as previously described [15,16] for 2 weeks, and cultures terminated for harvest of mRNA and immunohistochemistry studies as described above.

### Statistical analyses

Standard statistical analyses were performed utilizing InStat (GraphPad Software, San Diego, CA, USA). Correlation analyses and means ± standard deviations (SD) were calculated. *P *= 0.05 was considered to be the level of significance.

## Results

In all, 19 discs from 15 subjects were examined with immunohistochemical procedures to localize asporin. Table 1 summarizes the lumbar site and subject age and gender. The majority of the surgical disc specimens we receive come from grade III and IV discs. However, the present study does include four of the healthier grade I to II discs (mean subject age 35.3 years), and two of the most degenerate grade V (most degenerate) discs (mean subject age 38.5 years). Mean age for the six grade III discs was 45.5 years, and 53 years for the five grade IV discs.

**Table 1:** Demographic features for specimens studied with immunocytochemistry

Subject	Age (years)/gender	Site	Thompson grade	Other information
1	45/F	L4 to 5	1.5	CHTN: unknown
1	45/F	L3 to 4	II	CHTN: unknown
2	21/M	L5 to S1	II	Surgical specimen
3	40/M	L4 to 5	2.5	CHTN: MI
4	33/F	L1 to 2	III	CHTN: PE
5	46/F	L4 to 5	III	Surgical specimen
6	53/M	L3 to 4	III	Surgical specimen
7	29/F	L5 to S1	III	Surgical specimen
8	54/M	L4 to 5	III	Surgical specimen
9	58/M	L2 to 3	III	Surgical specimen
4	33/F	L3 to 4	IV	CHTN: PE
10	59/F	L3 to 4	IV	Surgical specimen
10	59/F	L1 to 2	IV	Surgical specimen
10	59/F	L2 to 3	IV	Surgical specimen
11	78/M	L3 to 4	IV	Surgical specimen
12	56/F	L2 to 3	IV	Surgical specimen
13	39/F	L4 to 5	IV	Surgical specimen
14	44/F	L5 to S1	V	Surgical specimen
15	39/M	L5 to S1	V	Surgical specimen

CHTN, Cooperative Human Tissue Network; F, female; L, lumbar; M, male; MI, myocardial infarction; PE, pulmonary embolism; S, sacral.

### Asporin immunolocalization

The first portion of our study of asporin and the intervertebral disc tested for the presence of asporin in human and sand rat discs using immunohistochemistry. Localization was seen within disc cells, but not in the extracellular disc matrix.

**Figure 1:**
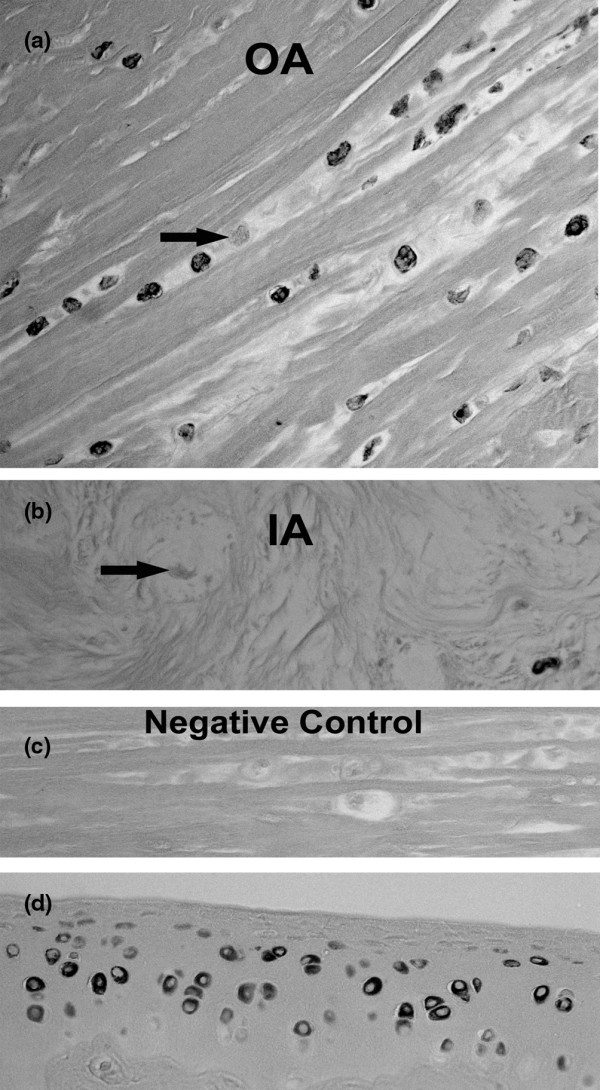
Presence of asporin. **(a) **Localization of asporin in the outer annulus of the human disc. Note that there are some cells that do not show asporin localization (arrow). **(b) **The presence of asporin is shown in the inner annulus of the human disc; note a nearby cell without asporin content (arrow). **(c) **Negative control. **(d) **Positive control showing localization of asporin in articular chondrocytes of the rat humerus (a, b, c × 260; d × 300).

**Figure 2:**
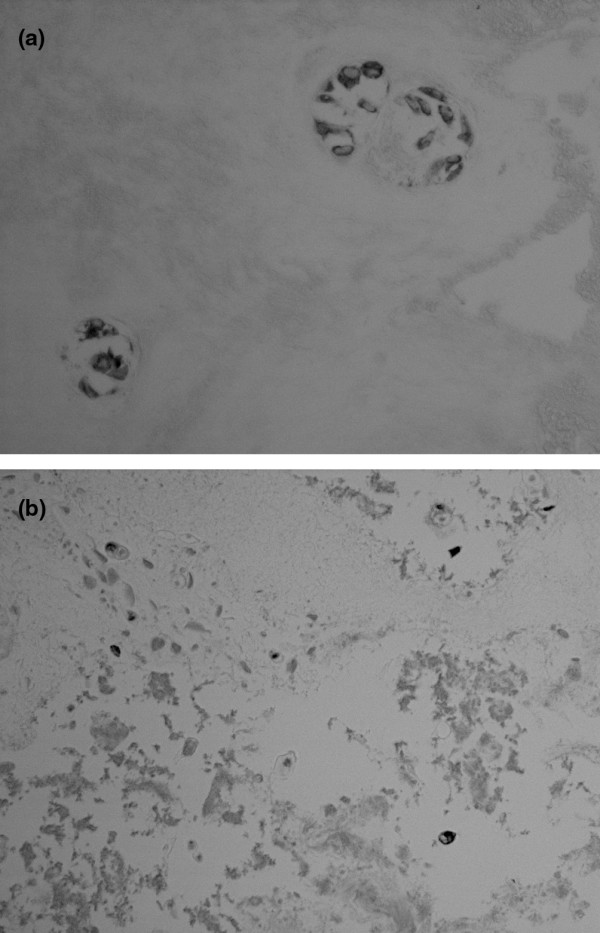
Cells with and cells without the presence of asporin are shown here in clusters in the inner annulus (**(a)** × 470). Rare cells show immunolocalization of asporin in the nucleus pulposus (**(b) **× 230).

**Figure 3:**
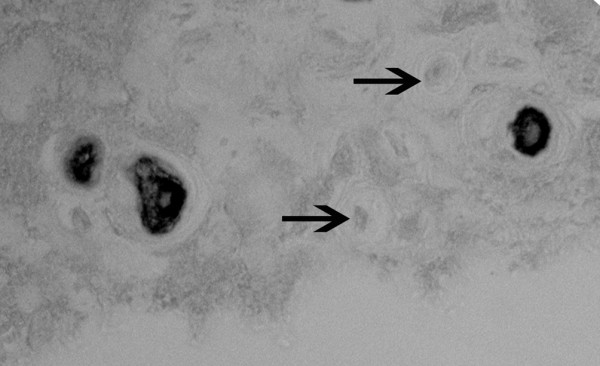
Cells with concentric rings of extracellular matrix in the inner annulus illustrated here show the presence or absence (arrow) of asporin (× 820).

As shown in Figure 1 for the human outer annulus, many cells show positive cytoplasmic immunolocalization of asporin. Some adjacent cells, however, did not show the presence of asporin (Figure 1a, arrow). Similar findings were seen in the more sparse cell population of the inner annulus as illustrated in Figure 1b. Cells present in clusters in the inner annulus were also studied; as noted in other disc areas, some, but not all, cells were positive for asporin immunolocalization (Figure 2a). In the nucleus pulposus, rare positive cells were present (Figure 2b). In the inner annulus, both positive and negative localization was present in cells that had concentric layers of extracellular matrix surrounding them (Figure 3).

**Figure 4:**
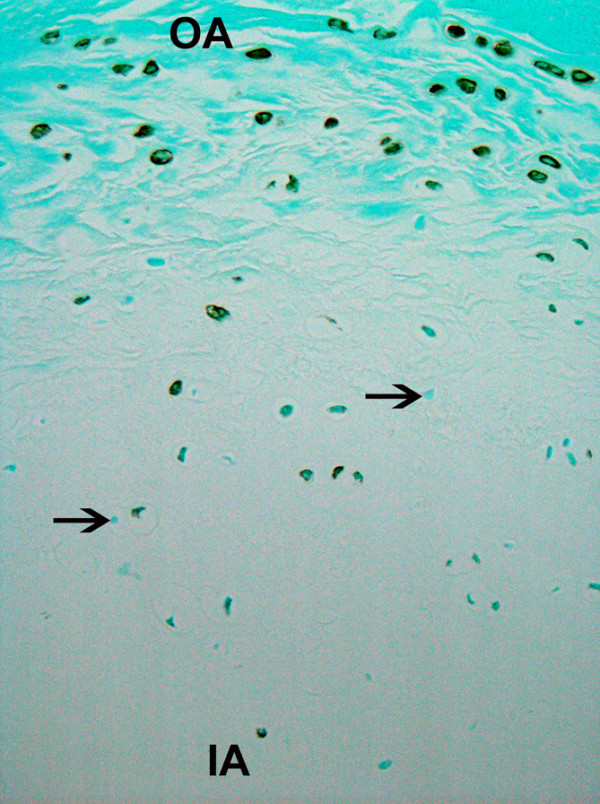
Sectioned en face, this sand rat lumbar vertebral specimen shows similar asporin localization to that seen in the human disc. The greatest asporin presence is in the outer annulus, with fewer cells showing asporin content in the inner annulus (arrows mark cells negative for asporin immunolocalization) (× 360).

The sand rat disc was also examined for asporin localization in sections of disc cut *en face*. Patterns of immunolocalization for the presence of asporin were similar to those seen in the human disc: the greatest localization was present in the outer annulus, with fewer cells positive in the deepest region of the inner annulus and nucleus pulposus (Figure 4).

**Figure 5:**
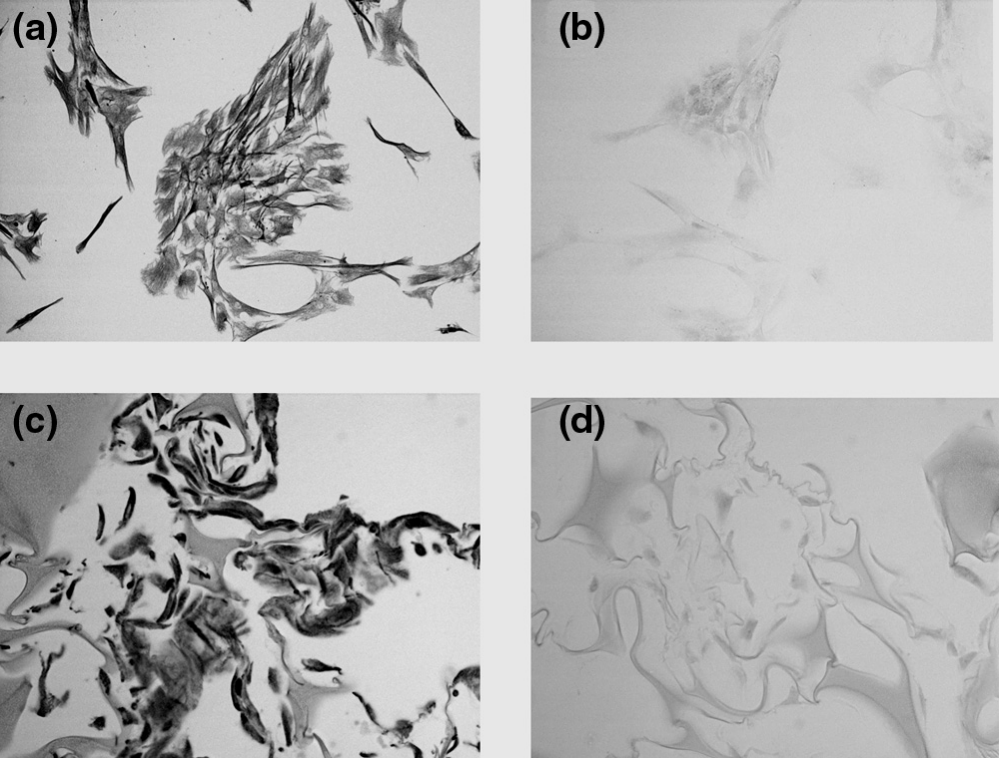
Immunolocalization of asporin *in vitro*. First panel: **(a) **annulus cells in monolayer show uniform localization; **(b) **monolayer negative control; **(c) **three-dimensional cultured annulus cells also show expression of asporin in all cells; **(d) **three-dimensional culture negative control. (a and b, × 66; c and d, × 275).

The presence of asporin was also detected *in vitro *in monolayer cultured human annulus cells (Figure 5a), and in annulus cells cultured in a three-dimensional collagen sponge (Figure 5c), which more closely mimics the *in vivo *microenvironment. Cells in monolayer and three-dimensional culture all showed positive asporin localization. (Figure 5b,d shows monolayer and three-dimensional culture negative controls, respectively).

### Asporin gene expression in human discs *in vivo *and *in vitro*

In the second part of our study of asporin and the intervertebral disc, asporin gene expression was evaluated in human disc tissue and in human annulus cells cultured in three-dimensional culture and in monolayer.

**Figure 6:**
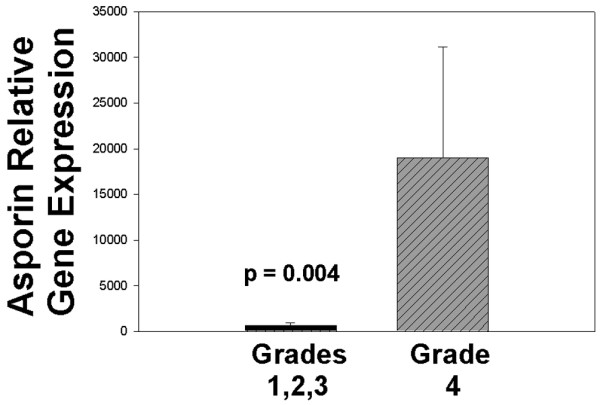
Asporin expression levels were significantly greater in more degenerate Thompson grade 4 discs vs expression levels in healthier grade 1, 2, and 3 discs (*P *= 0.004).

mRNA from 15 subjects, mean age 44.9 years, was harvested using laser microdissection as previously described [14]. An average asporin relative expression value of 7,123 was present in these specimens (ranging from 105 to 43,268). No correlation was present with asporin expression levels and age. However, the relationship between asporin expression Thompson grades approached significance (*P *= 0.0516). Further analysis showed that expression levels in the most degenerate discs studied here (grade 4) were significantly greater than that seen in healthier grade 1, 2 and 3 discs (Figure 6) (*P *= 0.004).

Gene expression was also analyzed in annulus cells cultured in three dimensions. These cells were derived from 13 human surgical specimens (mean subject age 45.6, range 23 to 72 years); subject demographic data are presented in Table 2. An average asporin relative expression value of 2,158 was present in these specimens (ranging from 135 to 6,251). No correlation was present between asporin expression levels and Thompson grade.

**Table 2:** Demographic features of subjects whose annulus cells were studied in three-dimensional gene expression analyses

Subject	Age (years)/gender	Site	Thompson grade	Other information
1	23/M	L5 to S1	I	Surgical specimen
2	45/F	T5 to T6	II	Surgical specimen
3	52/F	L2 to L3	III	CHTN: cardiac dysfunction
4	36/F	L5 to S1	III	Surgical specimen
5	31/M	L5 to S1	III	Surgical specimen
6	46/F	L2 to L3	III	Surgical specimen
7	28/F	L5 to S1	III	Surgical specimen
8	65/F	T12 to L1	IV	Surgical specimen
9	32/F	L5 to S1	IV	Surgical specimen
10	60/M	L4 to L5	IV	Surgical specimen
11	59/F	L3 to L4	IV	Surgical specimen
12	72/F	L4 to 5	IV	Surgical specimen
13	45/M	L5 to S1	V	Surgical specimen

CHTN, Cooperative Human Tissue Network; F, female; L, lumbar; M, male; S, sacral.

## Discussion

To date, although the D14 allele of asporin has been reported to be associated with lumbar disc degeneration in Asian subjects [10], there have been no studies on the location of asporin in the intervertebral disc, or on the levels of gene expression in disc tissue, in Caucasian subjects. Novel work reported here shows the presence of asporin associated with cells in the outer annulus and, less frequently, with cells in the inner annulus and nucleus pulposus. We also found that the asporin gene was expressed in healthy and degenerated discs, and by annulus cells in monolayer and three-dimensional cell culture.

Our studies showed significantly higher asporin expression levels in more degenerate intervertebral discs; these findings are similar to those presented from recent work by Kizawa *et al*. in cartilage from osteoarthritic subjects [4]. They found high levels in osteoarthritic subjects, but much lower levels in control subjects. They also found that a chondrogenic cell line with overexpression of asporin showed suppression of chondrocyte marker genes aggrecan and type II collagen during chondrocyte differentiation. These cells also showed suppression of TGFβ-mediated expression of aggrecan and type II collagen. Overexpression of asporin also was associated with proteoglycan content of the ECM. These findings led Kizawa *et al*. to suggest that asporin may play a major role in modulating chondrocyte matrix homeostasis.

Our studies presented here have several shortcomings that should be mentioned. Since the majority of the disc specimens which we received are Thompson grade III and IV, it is unfortunate that we did not have any of the most degenerate grade V specimens which could be used for gene expression studies. However, in spite of this problem, the data did show significantly greater asporin expression in the more degenerate grade IV specimens studied here as compared to expression in grades I, II and III (Figure 6, *P *= 0.004).

Secondly, we have not yet been able to examine our study population for the presence of the asporin polymorphisms now known to be present in Asian patients with disc degeneration [10]. Such studies are now underway. It is also important that further research be carried out at the cellular level to elucidate the function and role of asporin in the progression of disc degeneration. Final points for future work include studies of expression in nucleus pulposus cells. Another important future project would be to carry out an analysis in disc as was reported by Kizawa *et al*. [4] in cartilage. Another shortcoming is that we were not able to carry out a complete analysis of the localization of asporin in the aging/degenerating lumbar spine of the sand rat; we look forward to this future analysis.

## Conclusions

In the discs of Caucasian subjects studied here, and in the sand rat (an small rodent with spontaneous age-related disc degeneration), greater immunolocalization was found in the outer, compared to inner, annulus. Localization was rare in the nucleus. Similar patterns were found for the presence of asporin in lumbar discs of sand rats. Gene expression studies showed greatest expression of asporin in the more degenerate human discs *in vivo*. Asporin was also expressed in human annulus cells cultured in monolayer and three-dimensional culture.

## Abbreviations

*ASPN*: asporin gene; *CALM1*: calmodulin 1 gene; CHTN: Cooperative Human Tissue Network; ECM: extracellular matrix; *FRZB*: secreted frizzled-related protein 3 gene; GEO: Gene Expression Omnibus; SLRP: small leucine-rich proteoglycan; SNP: single nucleotide polymorphism.

## Competing interests

The authors declare that they have no competing interests.

## Authors' contributions

HEG and EN conceived the study and participated in design and coordination. HEG wrote the manuscript. YS and GH assisted with gene expression studies. YS assisted with a review of asporin in osteoarthritis. NZ and JAI performed histology and immunocytochemistry. All authors read and approved the final manuscript.
